# Diagnostic performance of CT and MRI in mandibular trauma assessment – a prospective comparative study

**DOI:** 10.1007/s10006-025-01496-3

**Published:** 2025-12-27

**Authors:** Adib Al-Haj Husain, Sameena Sandhu, Maximilian Eberhard Hermann Wagner, Egon Burian, Suen An Nynke Lie, Marc M. Precht, Daniel Zedler, Thomas Frauenfelder, Andreas M. Hötker, Bernd Stadlinger, Stefan Sommer, Peter Kessler, Harald Essig

**Affiliations:** 1 Department of Cranio-Maxillofacial and Oral Surgery, University Hospital Zurich, University of Zurich, Rämistrasse 100, Zurich, 8091 Switzerland; 2 Clinic of Cranio-Maxillofacial and Oral Surgery, Center of Dental Medicine, University of Zurich, Zurich, Switzerland; 3 Department of Cranio-Maxillofacial Surgery, GROW School for Oncology and Reproduction, Maastricht University Medical Centre, Maastricht, The Netherlands; 4 Diagnostic and Interventional Radiology, University Hospital Zurich, University of Zurich, Zurich, Switzerland; 5 Swiss Center for Musculoskeletal Imaging (SCMI), Balgrist Campus, Zurich, Switzerland; 6 Swiss Innovation Hub, Siemens Healthineers International AG, Zurich, Switzerland

**Keywords:** (MeSH): magnetic resonance imaging, Computed tomography, Mandibular fractures, Maxillofacial surgery, Black bone MRI, CT-like MRI

## Abstract

**Purpose:**

To compare the diagnostic performance of computed tomography (CT) and magnetic resonance imaging (MRI) for surgically relevant parameters in mandibular trauma, evaluating five in-house-optimized MRI protocols with a dedicated 15-channel mandibular coil.

**Methods:**

Fifteen patients with 30 acute mandibular fractures underwent 3T MRI using five optimized protocols: UTE, StarVIBE, Dark Bone, DESS, and STIR. Three independent observers qualitatively rated image quality, fracture line visibility, cortical delineation, and bone-to-soft-tissue contrast on a five-point visual analog scale (5 = best, 1 = lowest). Quantitative analysis included measurement of the maximum distance of the fracture gap. Descriptive statistics and inter-observer agreement (Krippendorff’s α) were calculated.

**Results:**

CT enabled rapid and accurate fracture detection in all cases. Among MRI protocols, UTE (85% of fractures correctly identified and accurately diagnosed) and StarVIBE (75%) demonstrated the best diagnostic performance, with excellent fracture line visibility, sharp cortical delineation, and strong inter-observer agreement (α = 0.7–1.0.7.0). Dark Bone and DESS provided variable results, while STIR was the least suitable for fracture depiction despite good soft-tissue contrast. Quantitative assessment of fracture gap distance showed the highest accuracy with StarVIBE, demonstrating minimal deviation compared to CT (0.13–0.21 mm; *p* > 0.05).

**Conclusion:**

While CT remains the standard in emergency settings and for delineating complex osseous pathologies, MRI, combining CT-like and water-sensitive sequences, offers a promising radiation-free alternative for simultaneous visualization of hard and soft tissues. This approach may facilitate comprehensive radiation-free trauma evaluation, improve detection of concomitant injuries, and further support individualized clinical decision-making.

**Trial registration number:**

Swiss National Clinical Trials Portal: SNCTP000006343, ClinicalTrials.gov ID: NCT07012850.

## Introduction

About one-third of all facial injuries involve mandibular fractures, ranking them among the most prevalent maxillofacial bone fractures [1]. This condition mainly affects young adult males, with interpersonal assaults, traffic accidents, and sports-related injuries identified as the main causes [1, 2]. Mandibular trauma often causes significant pain, compromises occlusion, and increases the risk of infections in affected individuals, thereby often requiring surgical intervention to reduce the risk of long-term complications [3].

Preoperative imaging with computed tomography (CT) and cone-beam computed tomography (CBCT), both considered reference standards, has become an integral part of the diagnostic trauma workup, enabling confirmation of suspected diagnoses from initial clinical examinations, detection of occult concomitant injuries, and providing precise guidance for surgical decision-making and planning [4]. In acute settings, particularly among polytrauma patients, CT is the imaging modality of choice due to its widespread availability, fast acquisition, and ability to provide high spatial resolution multiplanar reconstructions [5]. Nonetheless, its limitations include inadequate visualization of trauma-related soft tissue components and the cumulative radiation exposure, a concern particularly in the vulnerable younger trauma patients who often require repeated perioperative imaging [5, 6]. Thus, ongoing efforts aim to minimize or ideally eliminate radiation exposure in oral and maxillofacial trauma workflows, in accordance with the ALADAIP (As Low As Diagnostically Acceptable being Indication-Oriented and Patient-Specific) principle [7, 8].

In mandibular trauma, magnetic resonance imaging (MRI) is often considered a complementary modality to X-ray-based imaging for identifying associated inflammatory and traumatic soft tissue complications, such as temporomandibular joint (TMJ) involvement, muscle and nerve entrapment, and bone marrow edema [9]. Recent advancements, including the implementation of dedicated maxillofacial coils and optimized indication-specific MR sequences, such as Black Bone and CT-like MRI protocols, have overcome many limitations of traditional MRI [9–12]. These innovations enable simultaneous visualization of hard and soft tissues within short acquisition times. Despite these advances, few studies have directly compared CT and MRI for acute, surgically relevant parameters in mandibular trauma, including fracture line depiction, dislocation severity, and nerve involvement [13–15]. However, despite the frequent occurrence of mandibular trauma in young patients, the lack of comparative data across the broad spectrum of mandibular injuries using novel, fast-acquired CT-like MRI protocols limits progress toward radiation-free, clinical evidence-based imaging strategies.

Therefore, the aim of this prospective comparative study was to assess the diagnostic performance of CT and MRI for surgically relevant parameters in mandibular trauma by comparing five in-house-optimized MRI protocols in combination with a dedicated 15-channel mandibular coil.

## Materials and methods

### Study design

This prospective comparative study enrolled patients from routine clinical practice who presented to the emergency department, attended the outpatient clinic of the Department of Oral and Maxillofacial Surgery, or were referred by private practitioners between May and August 2025.

Inclusion criteria were: [1] acute mandibular trauma [2], age ≥ 18 years [3], no prior radiation-based imaging for the current trauma episode, and [4] ability to provide informed consent. Exclusion criteria were: [1] pregnancy or lactation [2], known contraindications to MRI (e.g., pacemakers, certain implants) [3], clinically significant comorbidities affecting image quality (e.g., severe neurocognitive disorders) [4], inability to comply with MRI procedures or study protocol (e.g., language barriers, psychological disorders, dementia), and [5] concurrent participation in other clinical trials that could interfere with the imaging study.

All participants in this study underwent both CT and MRI examinations, performed by trained clinical staff and research personnel from the Departments of Radiology and Cranio-Maxillofacial and Oral Surgery.

### Ethical statement

Ethical approval for this clinical study was obtained from the Cantonal Ethics Commission of Zurich, Switzerland (2024–02307). Written informed consent was secured from all participants, in accordance with the Declaration of Helsinki and its later amendments.

### Image acquisition

**Fig. 1:**
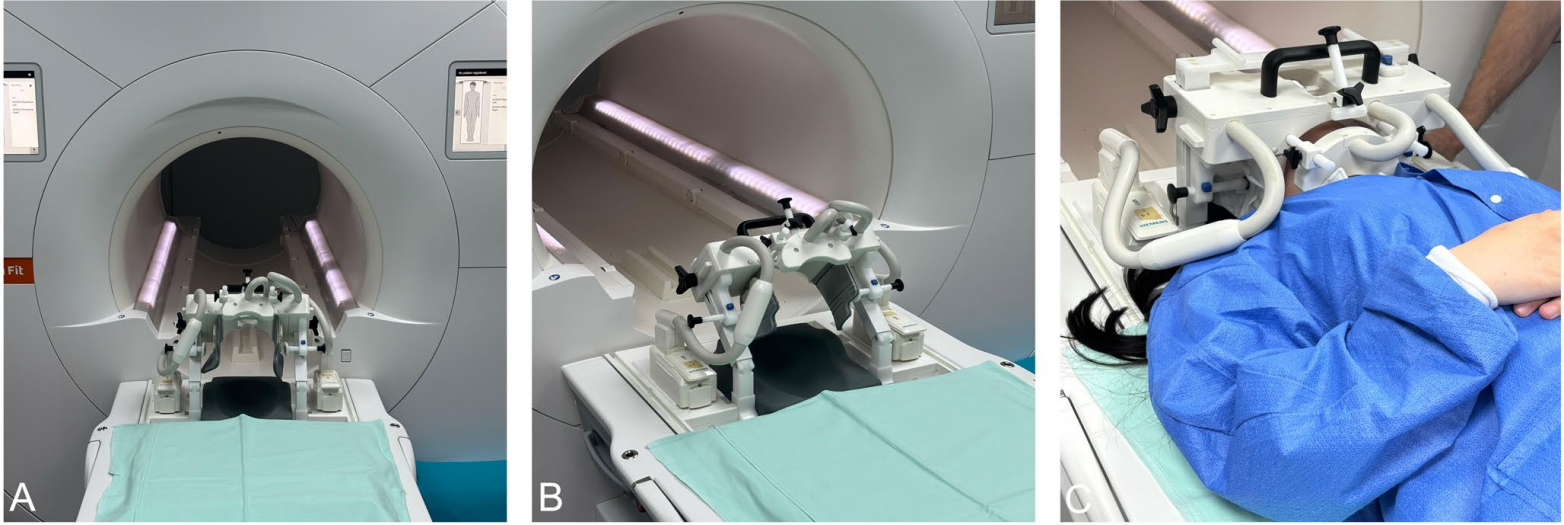
For magnetic resonance imaging (MRI) in the acute trauma setting, a 15-channel mandibular coil (NORAS MRI Products, Hoechberg, Germany) (**A** and **B**) was used on a 3 Tesla MRI system (MAGNETOM Vida^Fit^, Siemens Healthineers, Forchheim, Germany), providing a field of view of 32 x 16 x 16 cm. This coil incorporates a 14 + 1 receiver array integrated into a specialized, positioning system that can be adjusted to accommodate the individual anatomical variations of each patient (**C**). This adaptability enhances imaging precision, allowing for high-resolution visualization of the dentomaxillofacial complex and supporting effective MR-based trauma assessment

All enrolled participants underwent CT imaging according to the clinically established standard reference. In addition, each patient received an MRI scan on a 3 Tesla MAGNETOM Vida^fit^ system (Siemens Healthineers, Forchheim, Germany) using a dedicated 15-channel mandibular coil (NORAS MRI Products, Hoechberg, Germany) (Fig. 1). Five MRI protocols were acquired at sub-millimeter isotropic resolution: a research application 3D ultrashort echo time (UTE) protocol, 3D double-echo steady-state (DESS), a research application 3D T1-weighted Gradient Echo (GRE) “Dark Bone” protocol, 3D T1-GRE stack-of-stars volume interpolated breath-hold examination (StarVIBE), and 3D fast spin echo short-tau inversion recovery (STIR). The sequence parameters were as follows: UTE: repetition time, 4.6 ms; echo time, 0.04 ms; flip angle, 5 degrees; bandwidth, 1184 Hz/Px; fat suppression, none; Matrix 384 × 384 × 384; voxel size (acquisition), 0.6 × 0.6 × 0.6 mm^3^; acquisition time, 3:05 min, DESS: repetition time, 11.2 ms; echo time, 4.21 ms; flip angle, 30 degrees; bandwidth, 355 Hz/Px; fat suppression, water excitation normal; Phase encoding direction, R » L; Matrix read/phase 104 × 104; total acceleration factor, 2; voxel size (acquisition), 0.4 × 0.4 × 0.8 mm^3^; acquisition time, 6:41 min, Dark Bone: repetition time, 6.0 ms; echo time, 2.46 ms; flip angle, 4 degrees; bandwidth, 550 Hz/Px; fat suppression, standard; Phase encoding direction, R » L; Matrix read/phase 320 × 320; total acceleration factor, 2; voxel size (acquisition), 0.7 × 0.7 × 0.9 mm^3^; acquisition time, 3:13 min, StarVIBE: repetition time, 4.9 ms; echo time, 2.1 ms; flip angle, 9 degrees; bandwidth, 500 Hz/Px; fast fat saturation, standard; Phase encoding direction, R » L; Matrix read/phase 208 × 208; total acceleration factor, none; voxel size (acquisition), 0.7 × 0.7 × 0.5 mm^3^; acquisition time, 7:52 min, and STIR: repetition time, 3300 ms; echo time, 113 ms; flip angle, T2 var; bandwidth, 425 Hz/Px; fat saturation, standard; Phase encoding direction, L » R; Matrix read/phase 256 × 256; total acceleration factor, 4; voxel size (acquisition), 0.4 × 0.4 × 0.8 mm^3^; acquisition time, 9:57 min. Imaging data were initially acquired in either axial or coronal planes and reformatted through multiplanar reconstruction to generate views in other orientations.

### Image analysis

CT and MRI data were stored in DICOM format and analyzed using the local Picture Archiving and Communication System (PACS) in DeepUnity Diagnost (release v.1.1.1.2, Dedalus HealthCare, Bonn, Germany).

The imaging data assessments were performed by three independent observers from different medical specialties with varying levels of experience: Observer A (S.S.) is a board-certified attending in the Department of Cranio-Maxillofacial and Oral Surgery with 13 years of clinical experience; Observer B (E.B.) is a attending physician, board-certified radiologist and licensed dentist at the Institute of Diagnostic and Interventional Radiology with 10 years of experience in radiology, and Observer C (A.A.H.) is a resident in the Department of Cranio-Maxillofacial and Oral Surgery with 5 years of experience. A calibration meeting was held with the principal investigator and the observers to ensure consistency in image interpretation, aiming to standardize the assessment process under uniform viewing conditions. To minimize bias, all observers were blinded to the evaluations of others and to the MRI protocols, and all assessments were conducted in a randomized sequence.

### Qualitative analysis

The assessment of mandibular fractures involved determining their presence and exact anatomical localization, with both modalities employing the following scale for categorization: 0, pathology not identified; 1, pathology identified but inaccurately diagnosed; or 2, pathology identified and accurately diagnosed. Simultaneously, for each modality and imaging protocol, the evaluation time needed to achieve the correct classification, knowing that only mandibular trauma needed to be assessed, was recorded.

For each MRI protocol technical image quality was rated with regard to diagnostic confidence, background noise, resolution, artifacts using this modified five-point visual analog scale [13]: 5, excellent, with no restrictions for clinical decision-making; 4, very good, containing no substantial adverse effect for clinical decision-making; 3, average, borderline for clinical decision-making due to the image quality; 2, poor, substantial adverse effect for clinical decision-making; 1, very poor, not suitable for clinical decision-making.

Trauma-region-specific analysis included the evaluation of visibility of fracture lines, delineation of cortical borders, and bone-to-soft-tissue contrast for each protocol using a modified five-point visual analog scale [10, 14]: 5, excellent; fracture lines and cortical borders are sharply defined, optimal bone-to-soft-tissue contrast; 4, good; fracture lines and cortical borders are sharply defined with minor limitations; good bone-to-soft-tissue contrast; 3, fair; fracture lines and cortical borders are visible but less distinct with moderate limitations, intermediate bone-to-soft-tissue contrast; 2, below average; fracture lines and cortical borders are poorly defined, low bone-to-soft-tissue contrast; 1, poor; fracture lines and cortical borders cannot be defined, extremely low bone-to-soft-tissue contrast.

### Quantitative analysis

The maximum distance between the mandibular fracture margins was measured in the axial, coronal, and sagittal planes according to the method described by Feuerriegel et al. [14] and subsequently analyzed through protocol-specific inter-modality comparisons.

### Statistical analysis

Descriptive statistics were calculated, including the median, interquartile ranges (IQR), mean, and standard deviation. Inter-observer reliability was evaluated using Krippendorff’s alpha coefficient, with values interpreted according to established guidelines: a coefficient of 1.0 indicates perfect reliability, 0 represents agreement equivalent to chance, and values below 0 suggest systematic disagreement among observers [16]. Quantitative data were analyzed by calculating absolute and relative differences between CT measurements and each MRI protocol-specific measurement. Additionally, intermodal differences were systematically assessed using the Wilcoxon signed-rank test. All statistical tests were two-sided, applying a significance threshold of α = 0.05. All statistical analyses were performed using IBM SPSS Statistics (version 29.0.2.0, IBM, Chicago, IL, USA).

## Results

A total of 15 trauma patients (12 men, 3 women; median age, 23.6 years) with 30 mandibular fractures were enrolled in this prospective comparative study. Three independent observers assessed 15 standard-dose CT scans and 71 corresponding MRI reconstructions (four sequences missing due to non-completion of the scan), resulting in 86 image data sets per observer (Table 1).

**Table 1:** Patient characteristics, including demographics and trauma-related patho-anatomical characteristics, of all included study participants with acute mandibular trauma

Patient Characteristics		
N		15
Gender, male/female, N		12/3
Mean (SD) age at scan, years		30.7 ± 16.8
Median age at scan, IQR years		23.6 (19.1–38.4)
Age range, years		18–81
Total evaluated fractures		30
Fracture location	Right	24%
	Left	31%
	Bilateral	44%
Fracture pattern	Simple	80%
	Comminuted/Multiple	17%
	Displaced	17%
Fracture type	Symphysis	0%
	Parasymphysis	3%
	Body	17%
	Angle	14%
	Ramus	0%
	Condylar process	31%
	Coronoid process	3%
	Alveolar process and dental fractures	31%
Trauma mechanism	Traffic Accident	30%
	Assault	30%
	Fall	27%
	Sport-related Injury	13%
Time interval (in hours)	Trauma – CT	10.8 ± 16.2
	Trauma – MRI	90.09 ± 68.8
	CT – MRI	73.1 ± 62.5

### Qualitative results

The assessment of mandibular fractures, including their presence and precise anatomical location, was feasible in all cases using CT, with a mean evaluation time of 0:47 ± 0:18 min. In MRI, assessment times varied across protocols. The shortest evaluation times were observed for UTE (mean: 0:54 ± 0:22 min; 85% of fractures correctly identified and accurately diagnosed) and StarVIBE (mean: 0:55 ± 0:20 min; 75% correctly identified and diagnosed), whereas the longest times were required for the STIR protocol, with a mean of 1:07 ± 0:27 min (64% correctly identified and diagnosed).

Overall technical image quality was excellent for UTE (median 5, IQR: 5–5), and very good to excellent for DESS, StarVIBE, and STIR (each with a median 5, though with wider variability in observer ratings), while Dark Bone MRI showed slightly lower scores. Inter-observer agreement was strong to almost perfect across all protocols (α = 0.87 to 0.95).

Fracture line visibility was highest for UTE (median 5; narrow IQRs of 4–5 or 4.75–5.75) and StarVIBE (median 5; IQR: 2–5), with broader score ranges in DESS and Dark Bone, and STIR performing least well. Inter-observer agreement ranged from α = 0.83 to 0.90.

Cortical border sharpness was best in UTE, Dark Bone, and StarVIBE, with DESS and STIR showing lower but acceptable ratings. Bone-to-soft-tissue contrast was rated from fair to optimal across all MR protocols, with UTE, StarVIBE, and STIR protocols attaining the highest ratings in nearly every instance (α = 0.86–1.0.86.0).

**Fig. 2:**
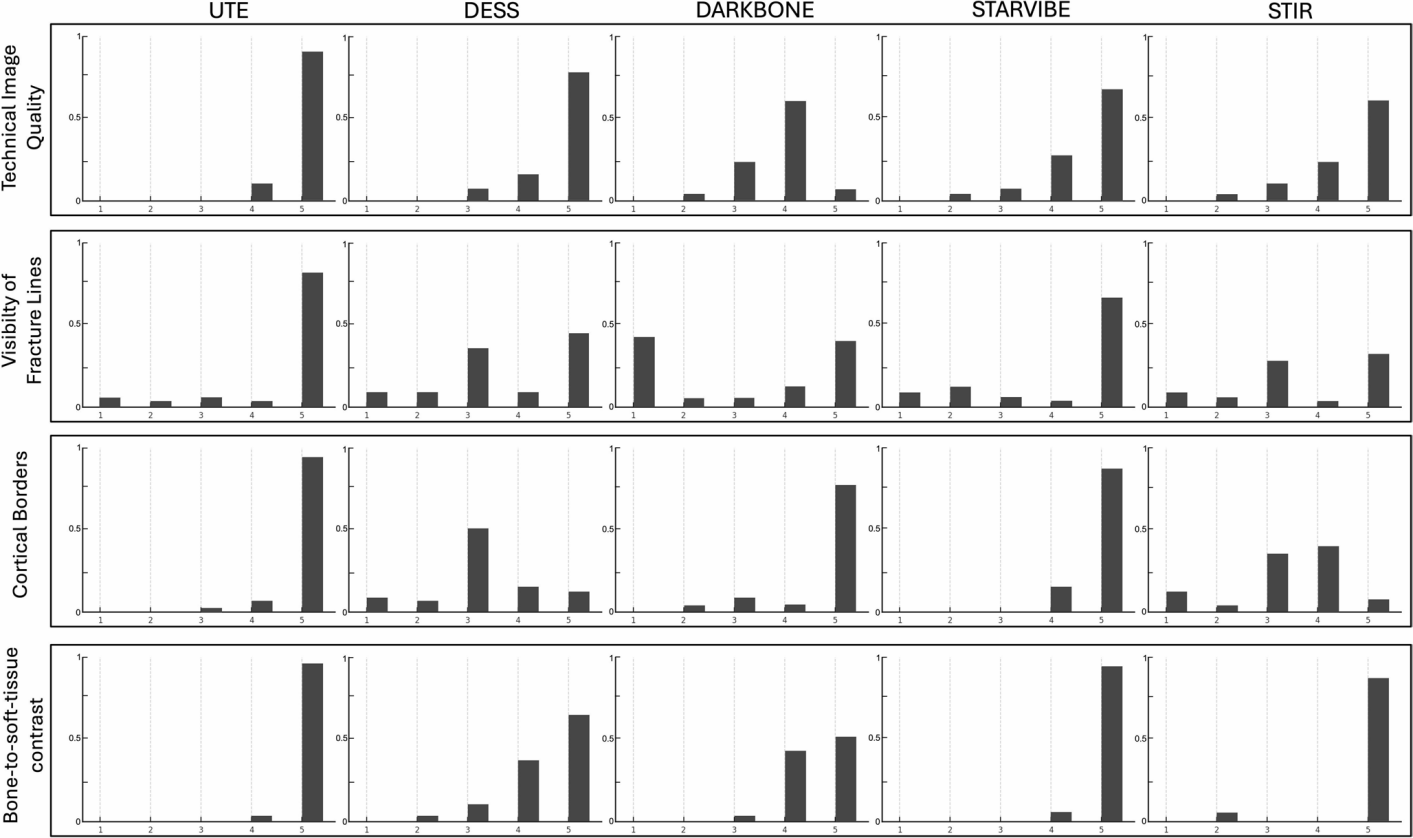
Frequency distribution of ordinal visual grading scores from qualitative assessments (technical image quality, fracture line visibility, cortical border delineation, and bone-to-soft-tissue contrast) for each MR-protocol (Rating: 5 = most favorable, and 1 = least favorable outcome)

Full details are summarized in Table 2, with the frequency distribution of visual grading illustrated in Fig. 2.

**Table 2:** Qualitative assessment of technical image quality, including diagnostic confidence, background noise, resolution, artifacts, as well as trauma-region-specific evaluation of fracture line visibility, cortical border delineation, and bone-to-soft-tissue contrast are reported by three observers (Observer A (senior oral and maxillofacial surgeon), B (senior radiologist), and C (resident in maxillofacial surgery) for each MR protocol (Rating: 5 = most favorable, and 1 = least favorable outcome). Results are presented as medians with interquartile ranges in parentheses. Inter-observer agreement is reported using krippendorff’s alpha coefficient [16]

	Imaging Protocol	Observer A	Observer B	Observer C	Krippendorff’s alpha
Technical Image Quality	UTE	5 (5–5)	5 (5–5)	5 (5–5)	0.9
	DESS	5 (5–5)	5 (4.5-5)	5 (5–5)	0.94
	DARK BONE	4 (3.25–5)	4 (3.25–5)	4 (3.25–4)	0.95
	STARVIBE	5 (4–5)	5 (4–5)	5 (4–5)	0.91
	STIR	5 (4.25–5)	5 (4.25–5)	5 (4.5–5)	0.87
Visibility of fracture lines	UTE	5 (4–5)	5 (4–5)	5 (4.75–5)	0.84
	DESS	4 (3–5)	4 (2.5–5)	4 (3–5)	0.83
	DARK BONE	4 (1–5)	4 (1–5)	4 (1–5)	0.9
	STARVIBE	5 (2–5)	5 (2–5)	5 (2–5)	0.96
	STIR	3 (2.25–5)	3 (3–5)	3 (2.5–5)	0.96
Cortical borders	UTE	5 (5–5)	5 (5–5)	5 (5–5)	0.7
	DESS	3 (3–4)	3 (3–3.5)	3 (3–4)	0.78
	DARK BONE	5 (5–5)	5 (5–5)	5 (5–5)	1.0
	STARVIBE	5 (5–5)	5 (5–5)	5 (5–5)	1.0
	STIR	4 (3–4)	3 (3–4)	4 (3–4)	0.88
Bone-to-soft-tissue contrast	UTE	5 (5–5)	5 (5–5)	5 (5–5)	1.0
	DESS	5 (4–5)	5 (4–5)	5 (4–5)	1.0
	DARK BONE	5 (4–5)	5 (4–5)	5 (4–5)	1.0
	STARVIBE	5 (5–5)	5 (5–5)	5 (5–5)	0.86
	STIR	5 (5–5)	5 (5–5)	5 (5–5)	1.0

*UTE* 3D ultrashort echo time, *DESS* 3D double-echo steady-state, Dark Bone: 3D T1-weighted Gradient Echo (GRE) “Dark Bone” protocol; StarVIBE: 3D T1-GRE stack-of-stars volume interpolated breath-hold examination; STIR: 3D fast spin echo short-tau inversion recovery

Interpretation of Krippendorff’s α: 1 = perfect reliability, 0 = no reliability beyond chance, <0 = systematic disagreement

**Fig. 3:**
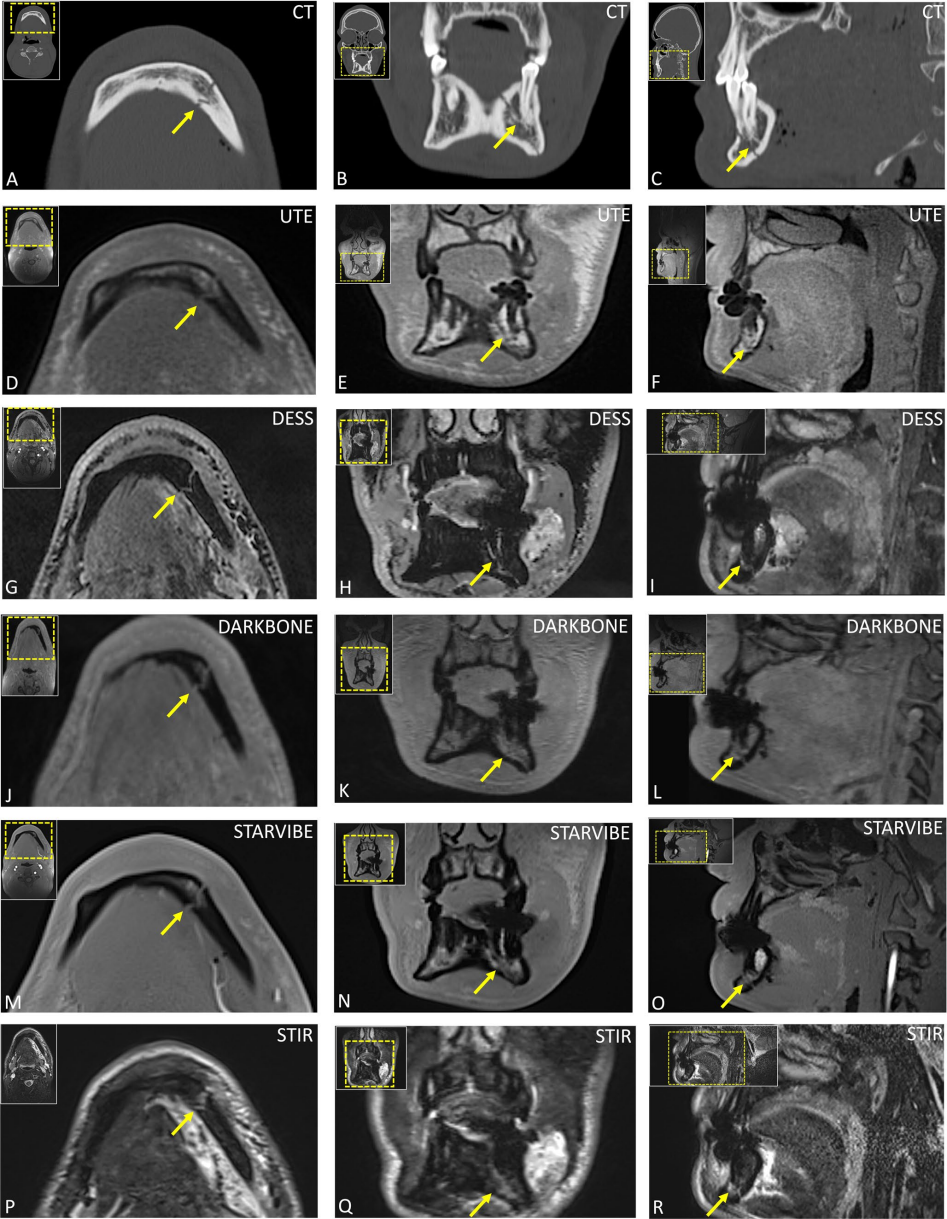
Open, double fracture of the mandible in a 22-year-old man following a physical assault, involving the right mandibular angle and the left paramedian region (teeth 32-33). Initial diagnosis was established with conventional computed tomography (CT) (axial, (**A**); coronal (**B**), sagittal (**C**)). The paramedian fracture component was primarily stabilized with maxillomandibular fixation (MMF) using an Ernst ligature at teeth 32 and 33. Subsequent magnetic resonance imaging (MRI) included five protocols (UTE (D-F), DESS (G-I), Dark Bone (J-L), StarVIBE (M-O), and STIR (PR)), depicting the fracture line and associated hematoma in axial (first vertical column), coronal (middle vertical column), and sagittal (third vertical column) reconstructions, with protocol-specific artifacts from the MMF illustrated

**Fig. 4:**
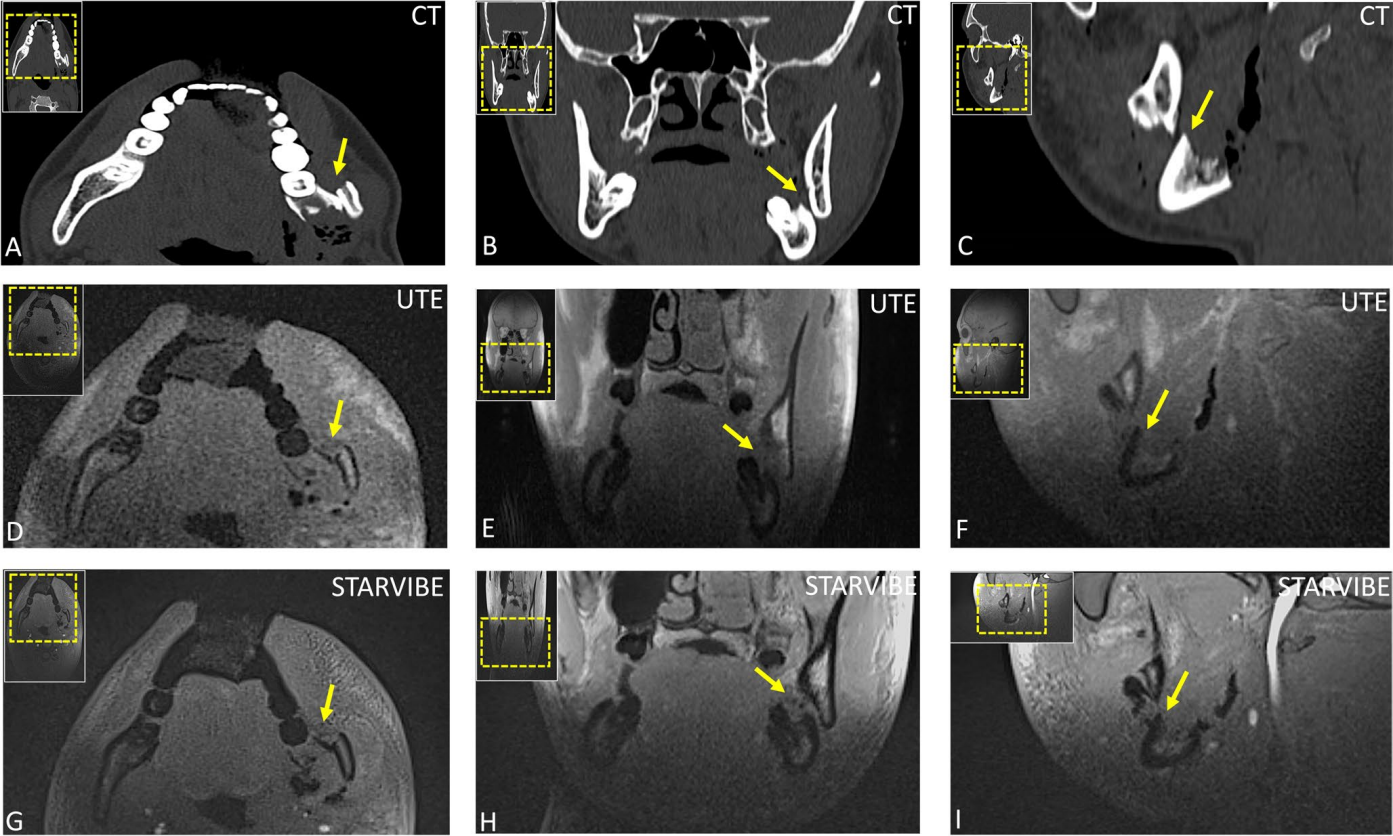
A multifragmentary fracture of the mandibular body at the level of the left mandibular angle (region 37-38) with displacement of approximately one mandibular width caudally (≈20 mm) and ≈11 mm medially, accompanied by a root fracture of tooth 38 (arrow) in an 18-year-old man following a collision during a football match. The injury is depicted in multiplanar computed tomography (CT) images (axial, (**A**); coronal (**B**), sagittal (**C**)) and in corresponding magnetic resonance imaging (MRI) sequences, including UTE (**D**-**F**) and StarVIBE (**G**-**I**)

**Fig. 5:**
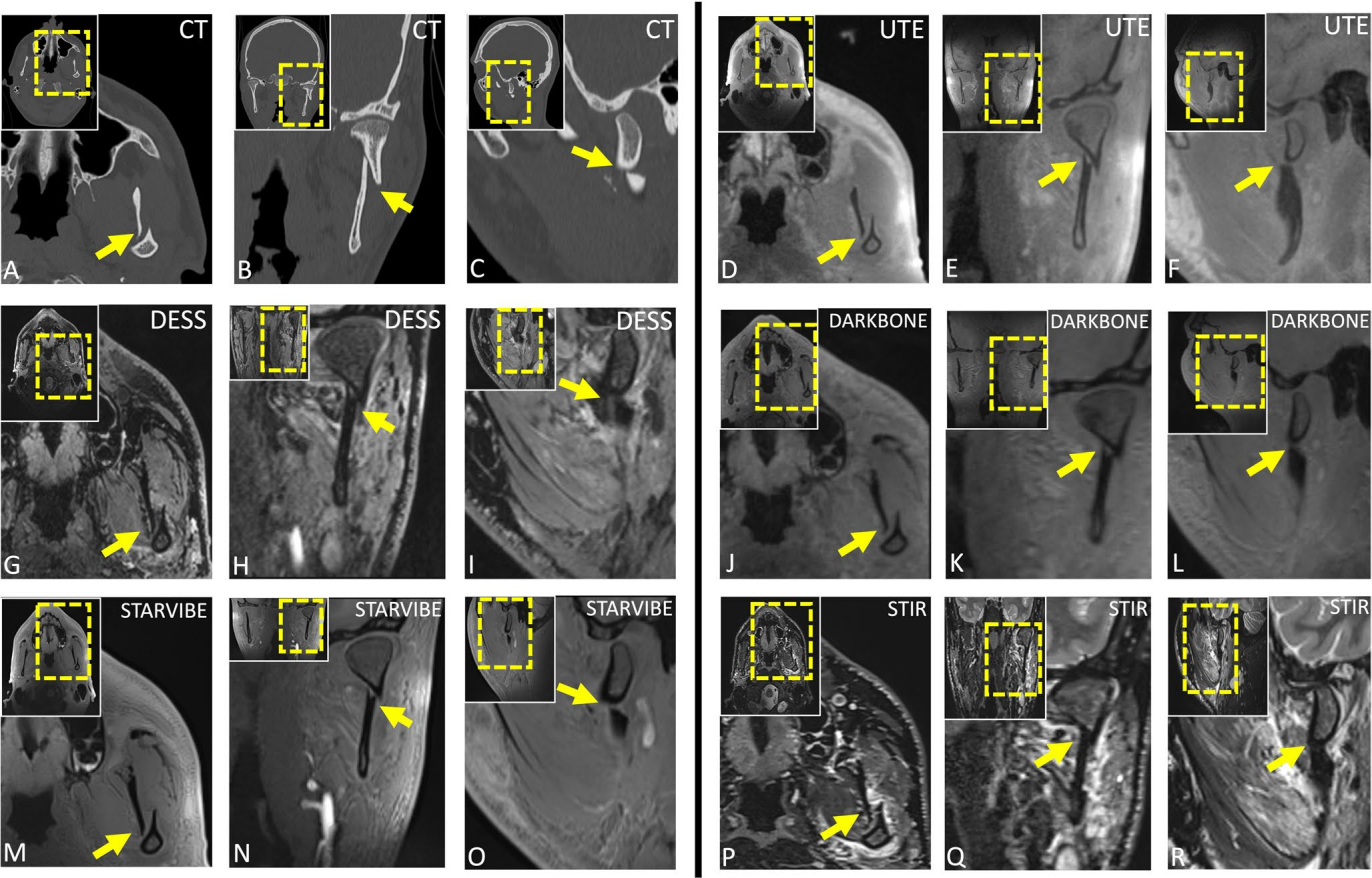
Preoperative imaging of a displaced left condylar fracture in an alcohol-intoxicated patient following a fall. Computed tomography (CT) images clearly delineate the fracture line (**A**-**C**), while corresponding magnetic resonance imaging (MRI) sequences (UTE (**D**-**F**), DESS (**G**-**I**), Dark Bone (**J**-**L**), StarVIBE (**M**-**O**), STIR (**P**-**R**)) provide detailed visualization of both osseous structures and surrounding soft tissues, revealing an edema in the masseter and medial pterygoid muscle

**Fig. 6:**
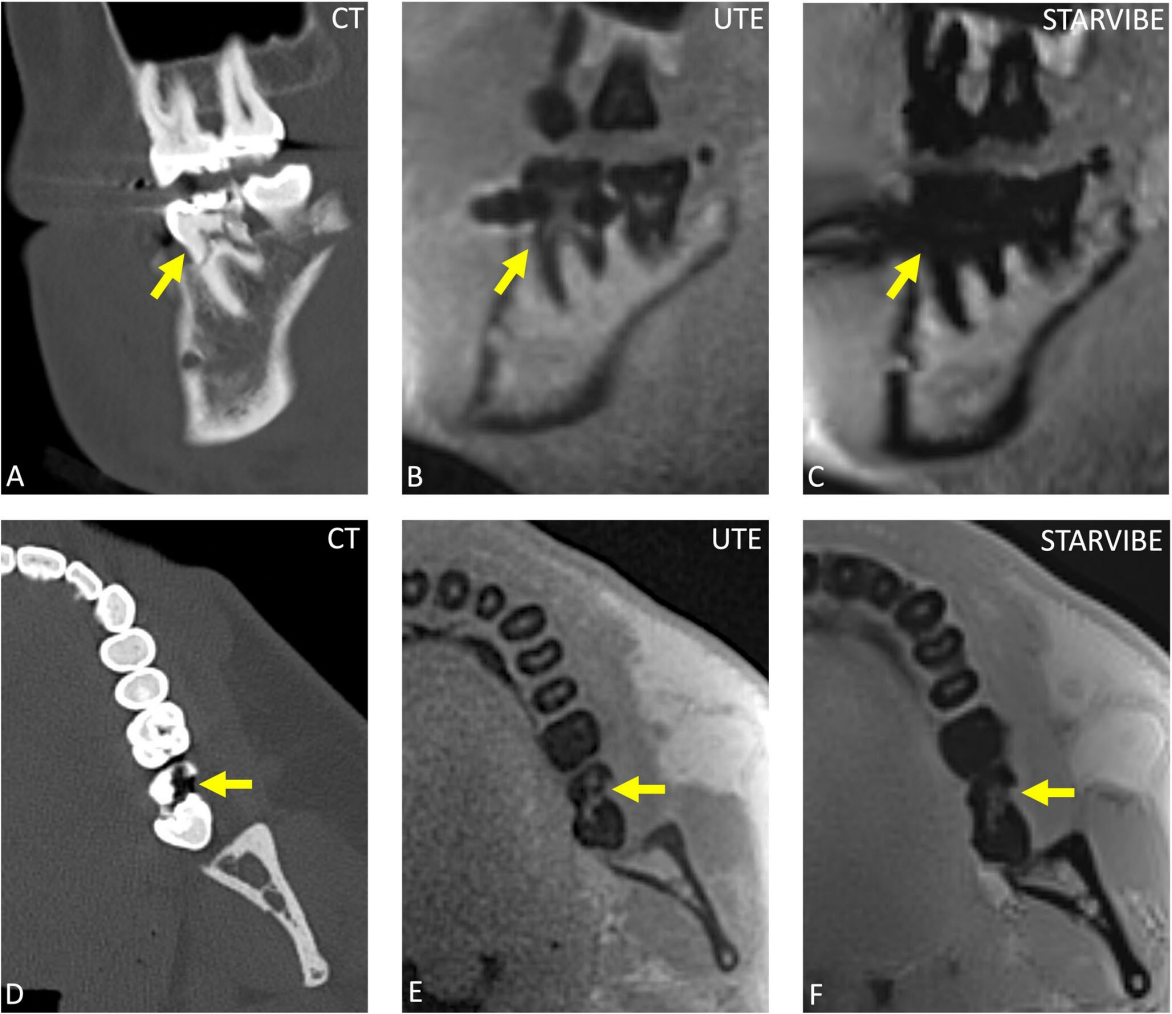
Visualization of dental trauma demonstrates a complex crown fracture of tooth 47 (**A**-**C**) and a fracture of tooth 37 (**D**-**F**), both excellently depicted on sagittal CT reconstructions (**A**, **D**). MRI provides limited visualization of dental cracks. Sagittal UTE reconstructions (**B**, **E**) show the fracture line of tooth 47 with good clarity but offer limited evaluation of the crown fracture. In contrast, sagittal StarVIBE reconstructions (**C**, **F**) provide worse visualization in both cases, not allowing for accurate diagnosis

Analysis by fracture localization (Table 3) revealed that UTE consistently achieved the highest scores across all trauma-related diagnostic parameters in every mandibular fracture subtype, with particularly strong performance in body, condylar process, and alveolar/dental fractures. StarVIBE also performed comparatively well, whereas DESS showed more variable results, receiving lower ratings for fracture depiction in body and alveolar process fractures. STIR achieved good technical image quality but was limited in fracture line visualization, especially in alveolar and coronoid process injuries as well as body fractures (Figs. 3, 4, 5 and 6).

**Table 3:** Analysis of image quality, fracture line visibility, cortical border delineation, and bone-to-soft-tissue contrast across mandibular trauma subtypes. Results are presented as medians with interquartile ranges in parentheses

	Imaging protocol	Parasymphisis	Body	Angle	Condylar process	Coronoid process	Alveolar process and dental fractures
Technical Image Quality	UTE	5 (5–5)	4 (4–5)	5 (5–5)	5 (5–5)	5 (5–5)	5 (5–5)
	DESS	5 (5–5)	5 (3–5)	5 (5–5)	5 (4–5)	5 (4–5)	5 (5–5)
	DARK BONE	4 (4–4)	4 (3–4)	4 (4–4.75)	4 (3–4)	4 (4–4)	4 (4–4)
	STARVIBE	4 (4–4)	4 (3–5)	5 (5–5)	5 (4–5)	5 (5–5)	5 (4–5)
	STIR	5 (5–5)	5 (3.75–5)	5 (5–5)	5 (5–5)	2 (2–2)	5 (5–5)
Visibility of fracture lines	UTE	5 (5–5)	5 (5–5)	5 (4.25–5)	5 (5–5)	5 (5–5)	5 (1–5)
	DESS	5 (5–5)	3 (3–5)	4 (3–5)	4 (3.5–5)	4 (4–4)	3 (2–3)
	DARK BONE	5 (5–5)	4 (1–5)	4 (1.5–5)	4.5 (3–5)	4 (4–4)	1 (1–2)
	STARVIBE	3 (3–3)	5 (4–5)	5 (2.75–5)	5 (3.25–5)	5 (5–5)	2 (1–5)
	STIR	3 (3–3)	3 (2–3.5)	4 (3–5)	5 (3–5)	1 (1–1)	2 (2–5)
Cortical borders	UTE	5 (5–5)	5 (5–5)	5 (5–5)	5 (5–5)	5 (5–5)	5 (5–5)
	DESS	3 (3–3.5)	3 (2–3)	3 (3–3)	3 (3–4)	4 (4–4)	3 (3–4)
	DARK BONE	5 (5–5)	5 (3–5)	5 (5–5)	5 (5–5)	5 (5–5)	5 (5–5)
	STARVIBE	5 (5–5)	5 (4–5)	5 (5–5)	5 (5–5)	5 (5–5)	5 (5–5)
	STIR	4 (4–4)	3 (2–3)	4.5 (4–5)	4 (3–4)	1 (1–1)	4 (3–4)
Bone-to-soft-tissue contrast	UTE	5 (5–5)	5 (5–5)	5 (5–5)	5 (5–5)	5 (5–5)	5 (5–5)
	DESS	5 (5–5)	4 (3–4)	5 (5–5)	5 (4–5)	5 (5–5)	5 (4–5)
	DARK BONE	4 (4–4)	4 (4–5)	5 (4.25–5)	5 (4–5)	5 (5–5)	5 (4–5)
	STARVIBE	5 (5–5)	5 (4–5)	5 (5–5)	5 (5–5)	5 (5–5)	5 (5–5)
	STIR	5 (5–5)	5 (5–5)	5 (5–5)	5 (5–5)	5 (5–5)	5 (5–5)

*UTE* 3D ultrashort echo time, *DESS* 3D double-echo steady-state; Dark Bone: 3D T1-weighted Gradient Echo (GRE) “Dark Bone” protocol; StarVIBE: 3D T1-GRE stack-of-stars volume interpolated breath-hold examination; STIR: 3D fast spin echo short-tau inversion recovery.

### Quantitative results

Quantitative measurements of maximum distances between mandibular fracture margins, compared with CT, demonstrated the smallest differences for StarVIBE (mean absolute difference of 0.13–0.21 mm across all planes), followed by UTE (0.27–0.46 mm). DESS (0.38–0.43 mm) and Dark Bone showed intermediate deviations (0.45–0.59 mm), whereas STIR consistently yielded the largest discrepancies (0.58–1.50 mm). The Wilcoxon signed-rank test revealed statistically significant differences for most protocols, except for StarVIBE reconstructions in all planes (*p* > 0.05) (Table 4).

**Table 4:** Quantitative evaluation of absolute and relative differences in radiological measurement differences (in millimeters (mm) and percentages (%)) of fracture gap or displacement between CT and MR protocols, with corresponding *p*-values from the Wilcoxon signed-rank test

	Imaging Protocol	Absolute Difference (mm) to CT (Mean ± SD; Median [IQR])	Relative Difference (%) to CT (Mean ± SD; Median [IQR])	*p*-Value
Axial	UTE	0.29 ± 0.42; 0.1 [0.1–0.43]	19.1 ± 31.6; 8.4 [0.94–15.8]	0.012
	DESS	0.4 ± 0.32; 0.4 [0.2–0.55]	24.77 ± 23.66; 20 [4.28–42.86]	0.001
	DARK BONE	0.45 ± 0.4; 0.3 [0.2–0.55]	23.97 ± 22.18; 20 [8.03–33.33]	0.001
	STARVIBE	0.13 ± 0.17; 0.1 [0.08–0.13]	7.77 ± 93; 5.41 [0.625–11.9]	0.054
	STIR	1.5 ± 3.79; 0.45 [0.4–1.13]	54.1 ± 63.23 [9.7-64.68]	0.001
Coronal	UTE	0.27 ± 0.43; 0.15 [0.1–0.3]	13.5 ± 17.6; 9.1 [0.81–15.26]	0.004
	DESS	0.38 ± 0.33; 0.3 [0.2–0.5]	24.86 ± 30.11; 10 [2.98–39.61]	0.001
	DARK BONE	0.59 ± 0.37; 0.5 [0.3–0.75]	28.5 ± 23.5; 20 [6.72–48.7]	0.001
	STARVIBE	0.15 ± 0.15; 0.15 [0-0.2]	10.6 ± 13.19; 4.37 [0-21.25]	0.4
	STIR	0.58 ± 0.39; 0.5 [0.33–0.7]	33.79 ± 37.39; 13.75 [7.41–56.74]	0.001
Sagittal	UTE	0.46 ± 0.51; 0.25 [0.18–0.55]	11.8 ± 9.08; 10.5 [5.15–20.56]	0.024
	DESS	0.43 ± 0.24; 0.2 [0.1–0.6]	13.86 ± 15.14; 10 [3.91–19.09]	0.048
	DARK BONE	0.5 ± 0.46; 0.4 [0.1–0.8]	14.93 ± 12.93; 10 [6.07–20.8]	0.004
	STARVIBE	0.21 ± 0.27; 0.1 [0-0.4]	9.74 ± 15.5; 2.78 [0-11.95]	0.398
	STIR	0.59 ± 0.36; 0.35 [0.2–0.88]	22.38 ± 28.15; 14.73 [4.69–29.64]	0.001

*CT* Computed Tomography, *UTE* 3D ultrashort echo time, *DESS* 3D double-echo steady-state; Dark Bone: 3D T1-weighted Gradient Echo (GRE) “Dark Bone” protocol; StarVIBE: 3D T1-GRE stack-of-stars volume interpolated breath-hold examination; STIR: 3D fast spin echo short-tau inversion recovery

## Discussion

This prospective study evaluated the diagnostic performance of five in-house optimized MRI protocols using a dedicated 15-channel mandibular coil in patients with acute mandibular trauma, compared with CT as the clinical reference standard. The findings indicate that advanced MRI techniques, particularly UTE and StarVIBE sequences, can accurately and reliably depict surgically relevant parameters in the assessment of mandibular trauma. When optimized for both hard- and soft-tissue imaging, the combination of dedicated protocols and the novel mandibular coil provides high image quality, excellent depiction of fracture lines, optimal bone-to-soft tissue contrast, and strong inter-observer agreement, highlighting MRI’s potential as a clinically feasible, radiation-free alternative to CT in the evaluation of mandibular trauma.

Growing evidence supports the feasibility and accuracy of MRI, particularly CT-like MRI using T1-weighted GRE sequences or UTE techniques, as both a complementary modality and a potential standalone alternative to CT for fracture detection across various medical fields [17–20]. In cranio-maxillofacial trauma, these studies have primarily focused on mandibular fractures, reporting diagnostic accuracies approaching those of CT [13–15]. To our knowledge, this is the first study to comprehensively evaluate all mandibular fracture subtypes in the acute setting, while also assessing a novel mandibular coil and directly comparing multiple MRI protocols.

In our study, CT provided a rapid and reliable assessment of all mandibular fractures, with the fastest evaluation times, averaging under one minute. MRI required longer interpretation, yet UTE and StarVIBE sequences approached CT in terms of efficiency, with mean reading times of approximately 55 s and diagnostic accuracies of 85% and 75%, respectively, supporting prior evidence of CT-like MRI performance [13, 14]. The STIR protocol required the longest evaluation times and demonstrated the lowest accuracy, making it less suitable for rapid fracture detection. Compared to earlier reports of near-perfect detection rates [14], our observed accuracies were lower, likely due to the broader and more anatomically complex spectrum of fractures included in our study.

Regarding image quality and susceptibility to artifacts, Burian et al. [13] and Feuerriegel et al. [14] reported that T1-GRE-based protocols (4.3/5) and a T1-FFE sequence (4.2/5) consistently achieved the highest ratings with minimal artifact burden. Slightly lower scores were noted for STIR (4.0/5) and DESS (3.9/5), all evaluated using a 5-point analog visual grading scale. Reported inter-observer agreement was very good across all assessments (κ = 0.79 to 0.87). These trends align with our results, in which UTE attained the highest median scores, while STIR and DESS demonstrated slightly higher image quality than previously reported, accompanied by even stronger inter-observer agreement across all protocols (α = 0.87–0.95). Furthermore, our analysis confirms that UTE and StarVIBE protocols provide the clearest depiction of fracture lines and sharply defined cortical borders across nearly all mandibular subregions, in line with previous evidence of the superior performance of CT-like MRI sequences for detailed bone assessment [10, 13, 14]. In contrast, DESS and STIR were less effective at accurately delineating pathoanatomical details of mandibular fracture lines. While fluid-sensitive sequences offer excellent soft-tissue contrast and highlight indirect fracture signs, our findings indicate that they are suboptimal as standalone protocols in acute mandibular trauma [10, 21]. For dental trauma, all protocols except UTE showed clear limitations, failing to achieve clinically sufficient standards in fracture depiction, in contrast to previously published ex vivo studies reporting comparable sensitivity and specificity to CBCT [22].

Confirming previous studies [14, 15], quantitative measurements of fracture dislocation showed excellent agreement between CT and MRI, particularly for StarVIBE, with mean absolute differences of less than 0.25 mm. While statistically significant intermodal differences were observed for the other protocols, the deviations in geometric accuracy were small and likely of no clinical significance.

The high diagnostic accuracy, rapid evaluation times, and strong inter-observer reliability, along with the excellent simultaneous visualization of hard and soft tissues at fracture sites provided by UTE and StarVIBE sequences in this study, further enhance and broaden the clinical role of MRI in the radiation-free assessment of acute mandibular trauma. The use of a dedicated 15-channel mandibular coil, tailored for close adaptation to the patient-specific mandibular anatomy [10, 23], enabled high-resolution isotropic imaging with accurate depiction of fracture lines, with the best-performing protocol (UTE) achieving acquisition times of just 3 min, effectively addressing key limitations of prior studies. From a clinical perspective, this study’s findings indicate that a single MRI protocol cannot yet fully replace CT for mandibular trauma assessment. However, combining a CT-like sequence (e.g., UTE) with a water-sensitive protocol can achieve adequate diagnostic accuracy in most cases, underscoring the importance of selecting a protocol based on clinical priorities. Especially in emergency settings, CT remains the fastest and most widely available modality; however, our findings demonstrate that optimized MRI protocols—particularly UTE and StarVIBE—allow rapid acquisition, supporting clinical workflow feasibility in acute but hemodynamically stable trauma cases. Moreover, MR-based trauma diagnostics may facilitate the detection of concomitant injuries, such as muscle entrapment, nerve involvement, or temporomandibular joint involvement, which may remain occult on CT [9, 13, 26]. This suggests that MRI could be integrated into the diagnostic pathway when radiation exposure is a major concern or when soft tissue assessment is needed concurrently. Nonetheless, it is essential to recognize that the most subtle and intricate fractures can still only be identified through CT imaging. Beyond the added diagnostic value of soft tissue assessment, this approach is especially advantageous for pediatric patients and young adults, a more radiosensitive population, as it minimizes cumulative radiation exposure from repeated perioperative imaging and reduces the risk of long-term radiation-induced adverse health effects [24, 25]. At the same time, it preserves all critical diagnostic information needed for safe and effective surgical planning.

Several limitations need to be mentioned. First, this study included a small sample of 30 mandibular fractures, which may limit the generalizability of the findings. Given the exploratory, feasibility nature of the study, no formal a priori power calculation was performed. Future studies with larger cohorts and appropriate sample size calculations are needed to validate these results across a broader spectrum of mandibular trauma. Second, while MRI acquisition times are clinically feasible, they remain longer than those of CT, which may limit their applicability in emergency situations for polytrauma patients. Therefore, future research should also examine cost-effectiveness, workflow integration, and potential effects on surgical decision-making and outcomes. Third, the time interval between CT and MRI, which in some cases extended up to 90 h, might have affected the assessments. Validation in patients undergoing imaging within shorter time intervals is therefore warranted.

## Conclusion

Optimized MRI sequences such as UTE and StarVIBE, when used with dedicated coils, provide diagnostic performance comparable to CT for mandibular trauma assessment. While CT remains the standard in emergency settings and for delineating complex osseous pathologies, MRI, combining CT-like and water-sensitive sequences, offers a promising radiation-free alternative for simultaneous visualization of hard and soft tissues, enabling comprehensive mandibular trauma evaluation and detection of concomitant injuries. However, a modality- and protocol-specific approach is essential, taking into account clinically relevant parameters, radiation exposure, and patient preferences to ensure optimal outcomes in comprehensive mandibular trauma management.

## Data Availability

The datasets analyzed during the current study are available from the corresponding author on reasonable request.

## Declarations

### Trial registration number

Swiss National Clinical Trials Portal: SNCTP000006343, ClinicalTrials.gov ID: NCT07012850, Registration date: May 2025.
